# Effects of Nutrient Limitation on the Synthesis of N-Rich Phytoplankton Toxins: A Meta-Analysis

**DOI:** 10.3390/toxins12040221

**Published:** 2020-04-01

**Authors:** Karen Brandenburg, Laura Siebers, Joost Keuskamp, Thomas G. Jephcott, Dedmer B. Van de Waal

**Affiliations:** 1 Department of Earth Sciences, Faculty of Geosciences, Utrecht University, Princetonlaan 8a, 3584 CB Utrecht, The Netherlands; 2 Department of Aquatic Ecology, Netherlands Institute of Ecology (NIOO-KNAW), Droevendaalsesteeg 10, 6708 PB Wageningen, The Netherlands; laurasiebers@hotmail.com; 3 Ecology & Biodiversity, Department of Biology, Utrecht University, Padualaan 8, 3584 CH Utrecht, The Netherlands; j.keuskamp@biontresearch.nl; 4 Biont Research, Abeelstraat 33, 3552 RC Utrecht, The Netherlands; 5 Faculty of Science, The University of Sydney, Camperdown, Sydney, NSW 2050, Australia; thomas.jephcott@sydney.edu.au

**Keywords:** harmful algal blooms, phycotoxins, eutrophication, stoichiometry, paralytic shellfish poisoning toxins, microcystin, cylindrospermopsin

## Abstract

Eutrophication has played a major role in the worldwide increase of harmful algal blooms (HABs). Higher input of key nutrients, such as nitrogen (N) and phosphorus (P), can stimulate the growth of harmful algal species in freshwater, estuarine, and coastal marine ecosystems. Some HAB-forming taxa, particularly several cyanobacteria and dinoflagellate species, are harmful through the production of N-rich toxins that have detrimental effects on the environment and human health. Here, we test how changes in nutrient availability affect N-rich toxin synthesis in cyanobacteria and dinoflagellates using a meta-analysis approach. Overall, N-rich toxin content showed an increase with P limitation, while it tended to decrease with N limitation, but we also observed substantial variation in responses both within and across genera and toxin groups. For instance, in response to N limitation, microcystin content varied from a 297% decrease up to a 273% increase, and paralytic shellfish poisoning (PSP) toxin content varied from a 204% decrease to an 82% increase. Cylindrospermopsin, produced by N_2_-fixing cyanobacteria, showed no clear direction in response to nutrient limitation, and cellular contents of this compound may thus vary independently of nutrient fluctuations. Our results confirm earlier reported stoichiometric regulation of N-rich phytoplankton toxins, showing increased toxin content with an increase in cellular N:P ratios, and vice versa. Thus, changes in N-rich toxin content largely follow the changes in relative cellular N content. Consequently, although nutrient limitation may limit bloom biomass and thereby bloom toxicity, our results warn that P limitation can cause accumulation of cellular toxins and thus lead to unexpected increases in bloom toxicity.

## 1. Introduction

Since the 1970s, the number of harmful algal bloom (HAB) outbreaks has increased dramatically worldwide [1,2]. This may partly be attributed to improved awareness and monitoring [2], but is largely caused by increased eutrophication of surface waters [1,3,4,5,6,7,8]. Since phytoplankton growth is often limited by key nutrients, such as nitrogen (N) and phosphorus (P), a higher supply of these nutrients to both freshwater and marine environments will lead to an increased build-up of algal biomass [9]. Moreover, changes in nutrient ratios can affect phytoplankton community composition, possibly favoring HAB-species, since phytoplankton species or groups can substantially differ in their preference for a type of nutrient regime, ratio, or form through differential physiological adaptations [4,10,11].

The formation of HABs adversely affects ecosystems, fisheries, tourism, and human health [10,12]. Some HAB species produce potent toxins that can accumulate in the food chain, which can lead to the death of fish, seabirds, and marine mammals, and thereby disrupt ecosystem structure and functioning [13,14,15,16]. Toxic HAB outbreaks in coastal waters are often caused by dinoflagellates, while HABs in freshwater environments are typically caused by cyanobacteria [17,18,19]. Toxins produced by both these phytoplankton groups can pose a risk to human health. For instance, dinoflagellate toxins can accumulate in shellfish and may cause severe shellfish poisoning syndromes upon the ingestion of seafood [20,21,22]. Cyanotoxins, such as microcystin, can cause acute liver failure, while chronic exposure to low concentrations through drinking water significantly increases the risk of liver and colorectal cancer [23,24,25]. Other symptoms of exposure to cyanotoxins may include, amongst others, abdominal pain, vomiting, diarrhea, skin irritation, weakness, sore throat, and headache [25,26].

The elemental composition of primary producers, such as phytoplankton, can vary substantially based on the relative availability of nutrients and light [27,28,29]. Nutrient availability strongly influences the production and composition of key biomolecules, such as fatty acids, amino acids, and nucleic acids [27,30,31]. Moreover, the production and composition of secondary metabolites, such as toxins, were also shown to follow stoichiometrically predictable patterns [32]. Numerous studies have already demonstrated the dependency of phytoplankton toxin production on nutrient availability [33,34,35,36,37,38]. Under N limitation, the production of N-rich toxins was shown to generally decrease, while P limitation caused accumulation of N-rich toxins following a relative excess of N. Next to bottom-up controls on phytoplankton toxin production; it should be noted that toxin content can also be significantly altered by grazers and other algae. For instance, *Nodularia spumigena* increased its nodularin content in the presence of eukaryotic microalgae, but not in the presence of copepods [39]. Moreover, several dinoflagellate species increased their toxin content when copepod grazers were present [40,41]. A variety of abiotic and biotic factors may thus alter phytoplankton toxin production in natural environments, but here in this study, we focus specifically on nutrient availability.

Although previous research has already demonstrated how N-rich toxin content depends on nutrient availability across phytoplankton phyla [32], the consistency of these responses across different genera and species remains unclear. Moreover, in recent years, an increasing number of studies have investigated the role of nutrient limitation on the regulation of phytoplankton toxins, most notably in cyanobacteria [42,43,44]. Therefore, we performed a meta-analysis to quantify the consistency in response of N-rich phytoplankton toxin content to N and P limitation across freshwater and marine phyla, genera, and species. Specifically, we tested whether phytoplankton toxin content increased or decreased under nutrient limitation using data from laboratory culture experiments. Toxins with a C:N ratio lower than the Redfield ratio (6.6) were considered N-rich, and include paralytic shellfish poisoning (PSP) toxins, microcystin, cylindrospermopsin, and nodularin (Table 1). We hypothesize lower amounts of N-rich toxins under N limitation and higher amounts of N-rich toxins under P limitation, based on earlier findings [32]. However, we also expect that these responses will be contrasted in cyanobacteria that can fix N_2_, as this process may compensate for N limitation, preventing a decrease in N-rich toxin content, while it involves P-related costs (enzyme synthesis), leading to a decrease in N-rich toxin content under P limitation.

## 2. Results

**Figure 1:**
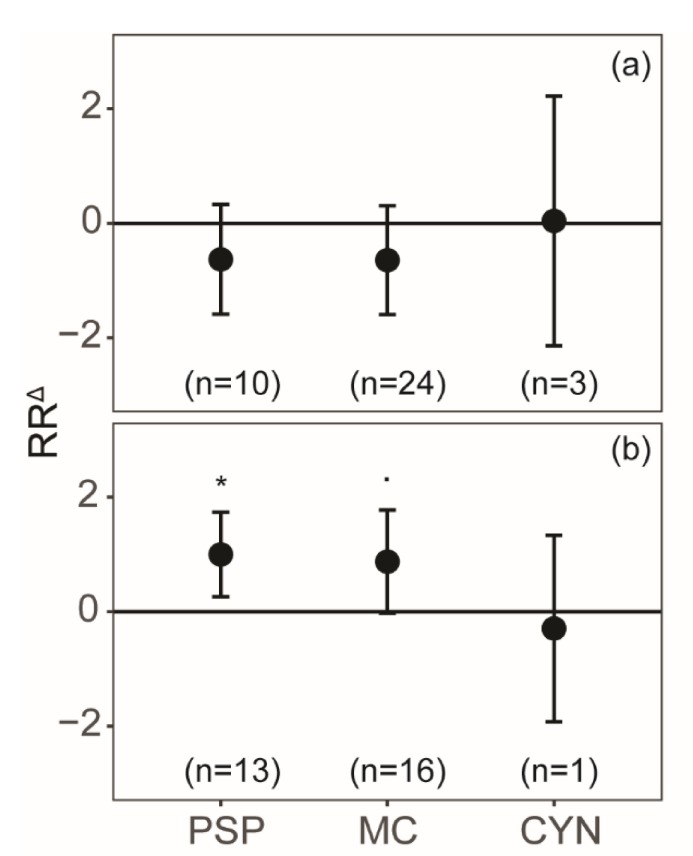
The natural log response ratios (RRΔ) for the different N-rich toxin contents, paralytic shellfish poisoning (PSP) toxins, microcystin (MC), and cylindrospermopsin (CYN), with (**a**) N and (**b**) P limitations. Here, cylindrospermopsin was produced by *Raphidiopsis raciborskii*, microcystin by *Microcystis* sp., and *Planktothrix* sp. and PSP toxins by *Alexandrium* sp. and *Gymnodinium catenatum*. Error bars represent the 95% confidence intervals and asterisks indicate the level of significance (· *p* < 0.1, * *p* < 0.05).

Cellular PSP toxin and microcystin content tend to decrease with N limitation, although the effects were not significant (*p* = 0.20 and *p* = 0.18, respectively; Figure 1a). Both these toxin groups showed an increase in response to P limitation, where cellular PSP toxins increased by 100%, and microcystins by 88% (Figure 1b). Unfortunately, no data that matched our criteria on nodularin in response nutrient limitation was available (due to lack of within-study variation). Cylindrospermopsin content showed a large variation in responses to either N or P limitation, and neither response was significant (*p* = 0.97 and *p* = 0.70, respectively; Figure 1).

**Figure 2:**
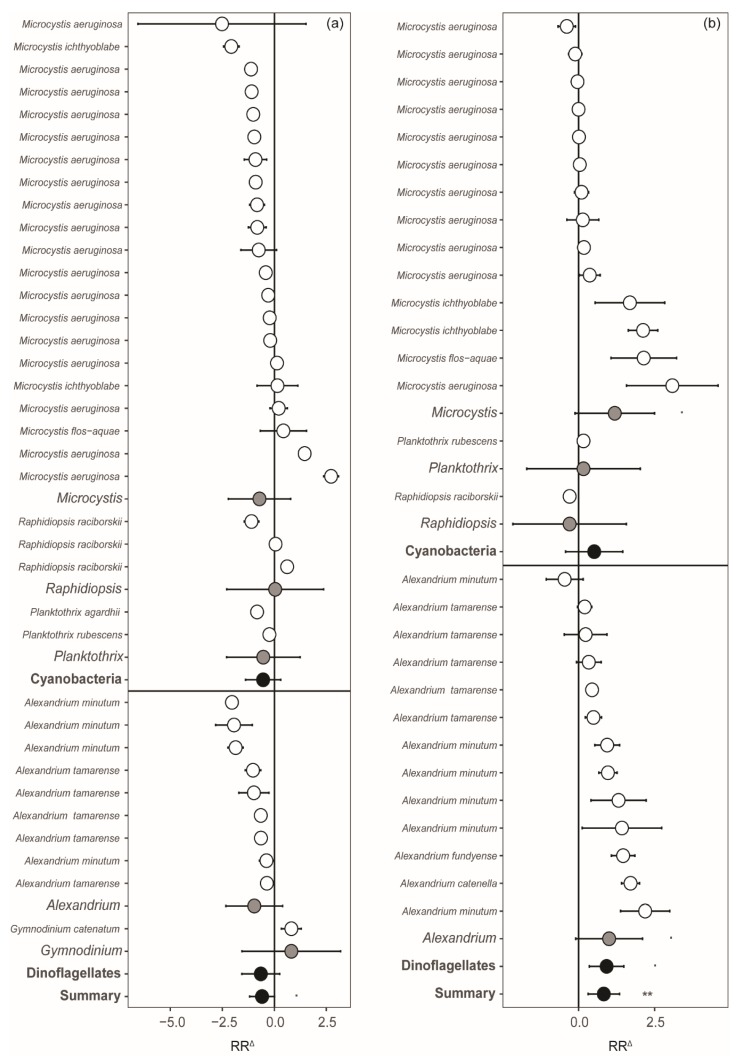
The natural log response ratios (RR^Δ^) for toxin content, shown for individual strains (white), different genera (grey), as well as the two phytoplankton groups (black), and a summarized response (black) with (**a**) N and (**b**) P limitation. Toxins produced by genera are indicated between brackets (MC = microcystin, CYN = cylindrospermopsin, PSP = paralytic shellfish poisoning toxins). Error bars represent the 95% confidence intervals and asterisks indicate the level of significance (· *p* < 0.1, ** *p* < 0.01).

The response of toxin contents to nutrient limitation varied across genera, species, and strains, most notably in cyanobacteria (Figure 2). Although most species and strains showed a decrease in N-rich toxin content with N limitation (Figure 2a), some did not respond at all, while three *Microcystis* strains, a *Raphidiopsis raciborskii* strain, and a *Gymnodinium catenatum* strain even increased their toxin content under N limitation. As a result, there was no significant decrease in N-rich toxins across species and genera, while N-rich toxins showed an overall decrease of approximately 60% across all phyla.

Comparable to N limitation, responses of N-rich toxin content to P limitation also varied substantially (Figure 2b). Most species and strains increased their toxin content under P limitation, resulting in an overall increase of 71%, while some species did not respond, and two *Microcystis aeruginosa* strains, a *R. raciborskii* strain, and an *Alexandrium minutum* strain, showed a decrease. We observed a significant increase in toxin content across *Microcystis* and *Alexandrium* species (i.e., a significant genera response) by 77% and 93%, respectively, as well as across dinoflagellate genera (i.e., significant phylum response) by 92%, while this was not the case for cyanobacteria.

**Figure 3:**
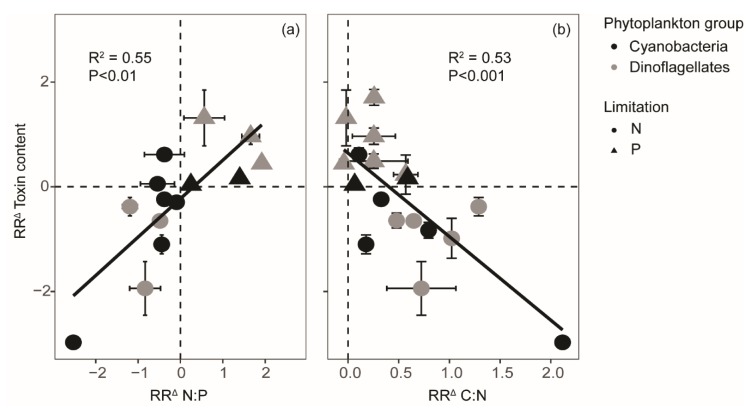
The natural log response ratios (RR^Δ^) for toxin content plotted against RR^Δ^ for cellular (**a**) N:P and (**b**) C:N ratios (n = 20). Error bars represent standard deviations.

We observed a clear relationship between the responses of N-rich toxin contents and cellular stoichiometry (i.e., changes in N:P or C:N ratios; Figure 3). Specifically, toxin content decreased together with N:P ratios when N was limited, and, vice versa, increased with N:P ratios when P was limited (R^2^ = 0.55, *p* < 0.01). Comparably, cellular contents of both toxins consistently decreased when C:N ratios increased under N limitation, while under P limitation, the toxin content increased but C:N ratios remained largely unaltered (R^2^ = 0.53, *p* < 0.001).

## 3. Discussion

In general, our findings confirm earlier studies and show that the cellular contents of N-rich toxins in phytoplankton are regulated by relative nutrient availabilities, with a decrease in response to N limitation and an increase with P limitation (Figure 2 and Figure 3). However, we also report substantial variation across strains, genera, and even phyla, as well as across toxins (Figure 1 and Figure 2).

The overall dependency of N-rich toxin content on N availability is in accordance with our hypothesis. Cells tend to contain less toxins, specifically microcystins and PSP toxins when N is limited. This suggests N is preferentially allocated towards population growth rather than the production of toxic secondary metabolites. Some of the variations in responses of N-rich toxins with N limitation may be explained by differences in the degree of N stress that the cells from the various studies have experienced. Indeed, more severe N limitation may be reflected by stronger reductions in cellular N:P ratios, or stronger increases in C:N ratios, which are followed by stronger decreases in toxin contents as well (Figure 3; see also [36]). Besides the overall nutrient status of a cell, levels of light or CO_2_ availability that affect C fixation and energy production also interact with the nutrient availability within the cell. For instance, higher light and CO_2_ availabilities may increase overall nutrient demands, and, when nutrients are limited, this may be followed by higher carbon:nutrient ratios and possibly an increase in the level of nutrient limitation experienced by the cells [45,46]. In contrast, relatively higher CO_2_ availabilities under N limiting conditions were shown to compensate for N limitation and led to a decrease in C:N ratios while PSP toxin contents increased [47]. Differences in light and CO_2_ availabilities not only result from differences in the supply of both resources but also depend on biomass build-up, where stronger biomass build-up is associated with lower light and CO_2_ availabilities through self-shading and CO_2_ fixation [38,46,48]. Consequently, differences in applied light and CO_2_ conditions, as well as differences in biomass build-up between the studies may lead to different responses in toxin contents as well. Although CO_2_ concentrations are often not reported, we could test for the confounding effect of light. Light varied from 5 up to 350 µmol photons m^−2^ s^−1^ across studies, yet this variation did not have a consistent effect on the response ratios (Figure S1).

Some species, particularly several cyanobacteria, increased their toxin content with N limitation (Figure 2a). Increases in microcystin content have been linked to the potential physiological role of these toxins to deal with severe N stress [49,50]. Various functions of microcystins have been postulated, including iron chelation, defense against grazers, and photosynthesis or other light related processes [51,52,53], as well as reducing oxidative stress [49,54,55,56]. During nitrogen starvation, reactive oxygen species might be formed through electron transfer from reduced ferredoxin to oxygen [57]. Production of microcystin would then be beneficial as it possibly protects cells under such adverse conditions. However, this does not appear to be a general strategy since, in most of the investigated studies, a decrease in microcystin content with N limitation was observed. Under N-limiting conditions, insufficient N may be available to effectively allow cells to deal with oxidative stress. However, this specific protective mechanism may involve the binding of microcystins to proteins [55]. With more cellular binding of microcystins to proteins, the cellular free microcystins (as measured in all publications) would decline. Therefore, cellular microcystin contents may not directly reflect shifts in synthesis, as they may also reflect a shift in allocation. Future experiments under N and P stress should, therefore, involve cell-bound microcystin analyses as well. Moreover, part of the produced toxins may also appear extracellularly, although this fraction might be low (e.g., <3% for microcystin) [38].

The absence of a response in the production of cylindrospermopsin with N limitation may be attributed to the N_2_-fixing properties of *R. raciborskii*. All strains included in our analysis performed N_2_ fixation when they were grown in N free medium, which possibly explains why they were still able to produce cylindrospermopsins [58]. N_2_ fixation is a costly process and cells can, therefore, exhibit lower growth rates under N limitation [59]. It seems, however, that the production of cylindrospermopsin is still evolutionarily favorable regardless of N availability and the cost for growth.

PSP-producing dinoflagellates almost all showed a decrease in toxin content with N limitation (Figure 2a). The N-richest amino acid arginine is a precursor in PSP toxin synthesis, and a decrease with N limitation can, therefore, be expected [33,60]. Although the exact function of PSP toxins is still debated, it likely plays a role in grazer deterrence [61,62,63]. Important intracellular functions that may be beneficial under N limitation, as for microcystin, have not been described for PSP toxins. The one *G. catenatum* strain included in our analysis increased its PSP toxin content under N limitation, which would suggest that PSP toxins might be functional to the cell. However, this increase may also be attributed to the experimental set-up, as cultures were inoculated with cells in the late stationary growth phase that were previously grown under N-replete conditions [64]. As the authors indicated, cells of *G. catenatum* may possibly have stored N under these non-limiting pre-experimental conditions, and cultures may therefore not have reached complete N deprivation [64].

Overall, more toxins were produced in response to P limitation (Figure 1b and Figure 2b), which is in line with previous findings [32]. Cell growth is hampered when P is limited, and cells may shunt excess N towards the production of secondary metabolites, such as toxins. Cyanobacteria may produce an effective N-rich storage compound, called cyanophycin, through luxury consumption [65,66]. Effective storage may possibly prevent N from being used to produce microcystin or cylindrospermopsin, and may thus explain why some cyanobacterial species showed a decrease in toxin content with P limitation (Figure 2b). In addition, reductions in toxin content were also linked to the reduced energy status of the cells as a result of P limitation [67]. Dinoflagellates showed a significant increase in toxin content with P limitation (Figure 2b), further supporting the hypothesis that toxins can accumulate in the cell in response to lower growth rates, given that the nutrients required for toxin synthesis are not limited [34,68]. PSP toxins are mainly produced in the G1 phase of the growth cycle, which can be prolonged in response to P limitation [69]. A single *A. minutum* strain did decrease its PSP toxin content with P limitation, but it should be noted that complete P deprivation may not have been reached in this particular experiment [70]. Moreover, we note that the P limitation dataset used here had a significant result when we tested for a possible publication bias. This implies that only studies with a strong significant result are published, while it may also reflect strong and consistent responses of phytoplankton N-rich toxin content to P limitation.

Responses of N-rich toxins to nutrient limitation were less consistent than previously reported [32]. This may be due to the inclusion of more data [42,43,44,71,72] and a more detailed analysis of variation across strains, species, and genera, providing a better reflection of the possible intra- and interspecific variation in toxin regulation with nutrient limitation. Our results also reveal some knowledge gaps with respect to N-rich toxin synthesis in response to nutrient limitation, in particular for cyanobacteria. One of the important traits of cyanobacteria is N_2_ fixation, but only a few N_2_-fixing cyanobacteria could be included in our analysis. Indeed, we lacked suitable data on common toxic N_2_-fixing species such as *Dolichospermum* sp., *Aphanizomenon* sp., and *Nodularia* sp., and our analysis thus precludes conclusion on this important functional group of cyanobacteria. Importantly, N_2_ fixers can also produce the N-containing toxins such as saxitoxin, nodularin, and anatoxin, next to cylindrospermopsin, but no studies were available that followed our criteria, thus revealing an overall lack in our understanding of the regulation of cyanobacterial toxins in response to nutrient limitation. Similarly, mixotrophic feeding strategies of dinoflagellates may also influence their respective toxin content independent of inorganic nutrient availability. Several toxic dinoflagellate species, including *Alexandrium*, were shown to be able to utilize organic substrates [73,74,75]. It has been proposed that their level of toxicity may depend on the presence of precursor amino acids in the organic feeding sources [76], although more research is needed to confirm this.

Phytoplankton toxicity can be altered by a variety of other factors besides nutrient availability. For instance, other abiotic environmental factors, such as temperature, light, salinity, and CO_2_ concentrations, can strongly influence toxin production [45,77,78,79,80,81]. In addition, toxin production is affected by the presence of grazers and other algae as well [39,40,41]. How phytoplankton toxin production will ultimately be affected by changes in nutrient availability will thus depend on the interplay with other environmental variables.

One of the most profound consequences of an increased nutrient loading in both freshwater and marine environments is the development of dense algal blooms [5,82,83]. This higher algal biomass in the water column is still an important predictor for total toxin concentrations, especially if the nutrient loads and ratios also select for toxic species [3,10,84]. Our results show, however, that bloom toxicity depends on specific nutrient ratios as well, since N-rich toxin synthesis can vary greatly between N and P limitation. Another factor that may influence bloom toxicity is the production of specific toxin analogs. Several analogs of the same toxin can exist that contain different amino acids or functional group constituents [85,86,87]. Dependent on the toxin group, more or less harmful analogs can be produced in response to N availability. For instance, more N-rich MC-RR was produced by *Microcystis* under high N conditions [67,88], which is the less toxic as compared to other microcystin analogs containing less N, like MC-LR [89]. Similarly, toxin composition also changed for PSP toxins produced by *A. tamarense* grown under different N:P ratios, where more toxic gonyautoxins were produced instead of C1 and C2 toxins with P limitation [90].

Changes in nutrient loading and nutrient ratios can greatly affect the magnitude and toxicity of HABs, which has major consequences for water quality. Here, we show that stoichiometric imbalances of N and P affect the production of N-rich toxins by freshwater and marine phytoplankton. Given the ongoing changes in nutrient loading and ratios through eutrophication, but also oligotrophication, our results may help to understand and predict how bloom toxicity can be altered in response to these shifts in nutrient availability, and would thus call for dual N and P removal [8,91].

## 4. Materials and Methods

### 4.1. Data Collection

We compiled a database containing data on cellular N-rich toxin content of harmful algal species under N and P limitation. The database includes only data acquired through single species culture experiments. Data were obtained by ISI Web of Science (https://www.webofknowledge.com/) searches using the query: (“phytoplankton” or “cyanobacteri*” or “dinoflagellate*”) and (“*toxin*” or “nodularin” or “microcystin” or “cylindrospermopsin”) and (“nitr*” or “phosph*”) and (“produc* or “synthesis”), yielding a total of 591 results on 18 April 2019. From these results, first titles and subsequently abstracts were reviewed, which led to a selection of 79 publications for screening. Datasets were considered suitable when they reported both nutrient-limited toxin content and high nutrient control. Studies with a small sample size (n < 3), or unreported sample size, were excluded from the analysis. After careful screening for suitability, 36 publications remained that contained 37 unique datasets for N limitation and 30 for P limitation (from both binary and gradational nutrient studies), which were included in our database. From these publications, we extracted the means and standard deviations of cellular toxin content under N limitation and a nutrient-replete control, and on P limitation and a nutrient-replete control, using Engauge data extraction software when needed [92]. In addition, information on experimental conditions (temperature, irradiance, light–dark cycle), type of N source (i.e., NO_3_^−^ or NH_4_^+^), cellular C, N, and P content, and whether the species was a diazotroph (i.e., N_2_-fixing) was extracted when available. The complete database is publicly available in Dryad under DOI: https://doi.org/10.5061/dryad.6m905qfww.

### 4.2. Response Ratios

For each unique dataset, log response ratios of cellular toxin content were calculated for paired observations of replete and limited N or P conditions. Values for cellular toxin content under nutrient-replete conditions were taken during the exponential growth phase for batch cultures or during steady state for light-limited semi-continuous or continuous cultures. For nutrient limitation, values for toxin content were used from the early stationary growth phase for batch cultures or at steady state for semi-continuous or continuous cultures, to ensure cells were nutrient-limited. Calculations of bias-corrected log response ratios (RR^Δ^) and variance were performed according to Lajeunesse (2015) [93]:

(1) $$RR^{\Delta }=\ln\frac{X_{limited}}{X_{replete}}+\frac{1}{2}\left[\frac{{\left(SD_{limited}\right)}^{2}}{n_{limited}*\;X_{limited}}-\frac{{\left(SD_{replete}\right)}^{2}}{n_{replete}*\;X_{replete}}\right]$$

(2) $$\begin{array}{cc} var\left(RR^{\Delta }\right) & =\frac{{\left(SD_{limited}\right)}^{2}}{n_{limited}\;*\;X_{limited}{}^{2}}+\frac{{\left(SD_{replete}\right)}^{2}}{n_{replete}\;*\;X_{replete}{}^{2}}\\ & \;\;\;\;\;\;\;\;+\frac{1}{2}\left[\frac{{\left(SD_{limited}\right)}^{4}}{n_{limited}{}^{2}\;*\;X_{limited}{}^{4}}-\frac{{\left(SD_{replete}\right)}^{4}}{n_{replete}{}^{2}\;*\;X_{replete}{}^{4}}\right] \end{array}$$

where *X* represents the mean toxin content, *SD* the standard deviation, and *n* the sample size.

### 4.3. Statistical Analyses

Statistical analyses were performed in R version 3.5.2 [94]. In order to calculate the overall natural log response ratio (RR^Δ^), mixed effect models were fitted to the dataset, yielding specific response ratios and their variances using the function rma.mv (package “metaphor” version 2.0-0) [95]. To correct for the dependency of experiments carried out within the same study and/or on organisms from the same genus and/or species, the factors reference, genus, and species were modeled as random effects. To attain response ratios per phytoplankton group, separate runs of the model were analyzed using the phytoplankton group as a moderator.

In addition, natural log response ratios for N:P and C:N ratio were calculated for each observation, where data was available, to reflect the differences in stoichiometry between nutrient-limited and replete conditions. A linear model was subsequently fitted through the response ratios of toxin content and N:P or C:N ratios to assess the role of N availability on toxin synthesis.

## Figures and Tables

**Table 1:** The C:N ratios of N-rich toxins.

Toxin (Short Name)	(Full Name)	C:N Ratio
PSP	Paralytic shellfish poisoning toxins	1.5
CYN	Cylindrospermopsin	3.0
MC	Microcystin (MC-LR; MC-RR)	4.3
NOD	Nodularin	5.1

## Supplementary Materials

The following are available online at https://www.mdpi.com/2072-6651/12/4/221/s1, Figure S1: The natural log response ratios (RR^Δ^) for toxin content plotted against irradiance for (**a**) N and (**b**) P limitation.
