# Supplementary material for: Effects of Nutrient Limitation on the Synthesis of N-Rich Phytoplankton Toxins: A Meta-Analysis
Source: Toxins (Basel). 2020 Apr 1;12(4):221. doi: 10.3390/toxins12040221 (PMC7232484; doi:10.3390/toxins12040221)
Supplement: Supplementary file 1 [file toxins-12-00221-s001.pdf]

Supplementary Materials: Effects of Nutrient Limitation on the Synthesis of N-Rich Phytoplankton Toxins – a Meta-analysis

Karen Brandenburg, Laura Siebers, Joost Keuskamp, Thomas Jepchott and Dedmer Van de Wall

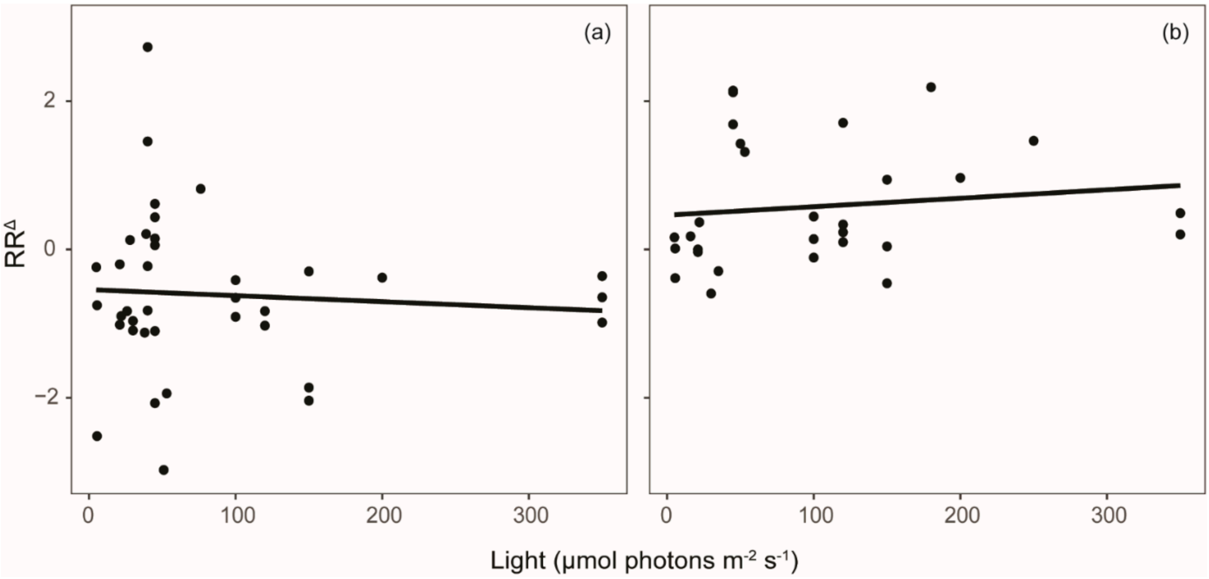

Figure S1. The natural log response ratios (RR$^{\Delta}$) for toxin content plotted against irradiance for (**a**) N and (**b**) P limitation. (**a**)R^2=-0.02, P=0.68, (**b**) R^2= -0.03, P=0.71.
